# Minimally Invasive (MINST) Versus Conventional Non‐Surgical Therapy for Residual Pockets in Patients With Periodontitis: A Randomized Clinical Trial

**DOI:** 10.1111/jre.70083

**Published:** 2026-06-18

**Authors:** Gaetano Isola, Alessandro Polizzi, Paolo Pesce, Angela Alibrandi, Giuseppe Pizzo

**Affiliations:** ^1^ Department of General Surgery and Medical‐Surgical Specialities, Unit of Periodontology University of Catania Catania Italy; ^2^ International Research Center on Periodontal and Systemic Health “PerioHealth” University of Catania Catania Italy; ^3^ Department of Surgical Sciences (DISC), Division of Prosthodontics and Implant Prosthodontics University of Genoa Genoa Italy; ^4^ Department of Human Pathology Azienda Ospedaliera Universitaria Policlinico “G. Martino” Messina Italy; ^5^ Department of Precision Medicine in Medical, Surgical and Critical Care University of Palermo Palermo Italy

**Keywords:** clinical trial, non‐surgical periodontal treatment, non‐surgical re‐instrumentation, periodontal treatment, periodontitis, pocket probing depth, residual periodontal pockets

## Abstract

MINST treatment was more effective in reducing median periodontal outcomes at 12‐month follow‐up than the conventional approach. Across 12‐months follow‐up, the overall PPD reduction was significantly influenced by a high number of cigarettes/day, erratic/non‐compliance supportive treatment, high no. pockets ≥ 4 mm BoP+, number of pockets 4–5 mm, number of pockets ≥ 6 mm, and by high FMBS.

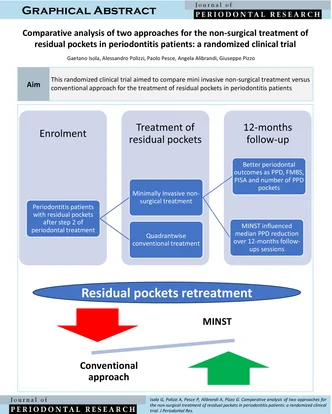

## 1 Introduction

Periodontitis is a chronic, multifactorial inflammatory disease of the periodontal tissues initiated by a pathogenic biofilm, leading to destruction of tooth‐supporting structures and eventual tooth loss if untreated [1]. According to the European Federation of Periodontology, treatment begins with step 1—patient motivation, oral hygiene instruction, and supragingival therapy—followed by step 2, which targets subgingival biofilm through conventional nonsurgical periodontal treatment (CNST) based on the patient’s risk profile [2, 3]. Despite these interventions, residual periodontal pockets frequently persist, particularly in deeper sites, and represent a major risk factor for disease progression and tooth loss. Re‐treatment of these residual pockets typically involves repeated subgingival instrumentation; however, evidence regarding its additional clinical benefit remains inconsistent [3]. Minimally invasive non‐surgical therapy (MINST), involving precise debridement with ultrasonic devices, mini curettes, and magnification under local anesthesia, has been proposed as an alternative approach. It may improve clinical outcomes by minimizing tissue trauma, reducing gingival recession, and enhancing healing while effectively controlling subgingival biofilm [4, 5].

The present two‐arm, superiority randomized controlled clinical trial aimed to compare CNST and MINST for the management of residual periodontal pockets over a 12‐month follow‐up. Additionally, predictors of probing pocket depth (PPD) reduction were evaluated to assess factors influencing treatment efficacy.

## 2 Methods

### 2.1 Study Design

Patients with periodontitis, diagnosed according to the established periodontitis definitions and re‐evaluated 12–15 weeks after Steps 1 and 2 of periodontal therapy [2], were enrolled for the present study and allocated to one of the two groups as CNST (control group) or MINST (test group) (Figure S1).

The study was conducted in observance of the Principles of the Declaration of Helsinki on experimentation involving human subjects. Written consent was obtained from all patients before the active stage of treatment and subsequently for the treatment of residual pockets. The study was reviewed and approved by the Institutional Review Board of the University of Catania (approval number: 24/20/PAR) and followed the CONSORT guidelines (Table S1). The study protocol was registered on clinicaltrials.gov (NCT06993519).

The subparagraphs sample size calculation, study participants, outcome measure, step 1 and 2 treatment, calibration and masking, randomization and statistical analysis are reported in the File S1.

### 2.2 Study Outcomes

The primary outcome evaluated was the median PPD reduction, recorded using a manual periodontal probe.^1^ Secondary outcomes included the evaluation of the clinical attachment level (CAL), the number of teeth present, the number of sites with a PPD ≥ 4 mm that were positive to bleeding on probing (BoP), and the number of PPD pockets, stratified for PPD depth.

### 2.3 Residual Pocket Treatment

All patients who still presented both suprabony or infrabony residual periodontal pockets after 12–15 weeks of follow‐up after step 2 treatment (study baseline) received an additional retreatment by the same operator with over 15 years of expertise (GI) that started with a new session of individualized oral hygiene instructions, which included interdental plaque control with interproximal brushes (tailored to each patient) and toothbrush using a modified Bass technique. All of the participants were provided with the same type of toothpaste,^2^ toothbrush, and interdental brushes.^3^

In the control group, treatment was performed through CNST using Gracey curettes (1/2, 5/6, 7/8, 11/12, and 13/14) and an ultrasonic device with inserts (No. #1, 2#, and #1S) only in experimental sites, according to the operator preference.^4^ Only sites that required retreatment were treated, per quadrant, in four separate sessions with a one‐week interval, starting from the right maxillary quadrant.

In the test group, MINST was performed, with the same protocol per quadrant as the control group, only on the experimental sites under local anesthesia through careful subgingival debridement using ultrasonic scalers with specific thin tips (Instrument PS^5^) and Gracey micro curettes (Mini Five curettes) to minimize soft tissue trauma [6]. The treatment protocol was performed using 3.5× loupes with head support through debridement of the supra‐ and subgingival deposits, starting the debridement from the bottom to the top of the periodontal pockets.

The clinical examination was performed at retreatment baseline and follow‐up intervals 1, 3, 6, 9, and at 12 months from baseline in which patients were recalled and periodontal parameters collected by one clinician not involved in the other phases of the study.

## 3 Results

### 3.1 Sample Characteristics

A final number of 54 stage III and IV periodontitis patients, 27 per group, were included for analysis and re‐treated for residual pockets (Table S2). None of the patients were lost during the follow‐up sessions, and no complications were noted.

### 3.2 Primary Outcome

The mean treatment duration was 9.2 ± 4.2 min for the CNST group and 10.5 ± 3.7 min for the MINST group (*p* = 0.112).

Compared to baseline, at 12‐month follow‐up, the MINST group showed better periodontal outcomes than the CNST group with respect to median PPD reduction (*p* = 0.026), FMBS (*p* = 0.039), and PISA (*p* = 0.048) and a reduction in the number (no.) of pockets PPD ≥ 4 mm (*p* = 0.004), no. pockets ≥ 4 mm BoP positive (*p* = 0.036), no. pockets PPD ≥ 6 mm (*p* = 0.007), and in the % PPD ≥ 4 mm (*p* = 0.012), while the other parameters were not different (Tables S3 and S4).

The mixed generalized linear regression analysis evidenced that the median PPD reduction over the follow‐up session was significantly influenced by the MINST treatment (crude, *p* = 0.014; adjusted, *p* = 0.023). Specifically, the adjusted estimated model also showed that the median PPD reduction was significantly impacted by a high number of cigarettes/day (*p* = 0.032), erratic/non‐compliance supportive treatment (*p* = 0.041), high no. PPD ≥ 4 mm BoP+ (*p* < 0.001), high no. PPD sites 4–5 mm (*p* = 0.031), no. PPD ≥ 6 mm (*p* = 0.027), (*p* = 0.025), and by high FMBS (*p* = 0.005) and PISA (*p* = 0.018) (Table S5), while the other variables resulted not significant (Table S6). The random effect variable, such as the variable patient, did not influence, in both crude and adjusted models, the reduction of median PPD at all follow‐up sessions (crude, *p* = 0.331; adjusted, *p* = 0.114), while the number of diseased sites significantly impacted the reduction of median PPD (crude, *p* = 0.045; adjusted, *p* = 0.048). In the adjusted model, the VIF was 2.44, indicating moderate multicollinearity and the reliability of the coefficient estimates.

## 4 Discussion

This randomized clinical trial evaluated the clinical efficacy of MINST compared with CNST for the treatment of residual periodontal pockets and MINST demonstrated superior improvements in several periodontal parameters, particularly PPD, BoP, FMBS reduction, and CAL gain, compared to CNST at 12‐months follow‐up.

Specifically, MINST group achieved significantly greater reductions in median PPD and CAL, as well as larger increases in the proportion of sites with PPD < 4 mm. In this regard, a recent systematic review [7] reported that, even with a significant heterogeneity between studies, the maximum effect of Step 2 therapy on PPD reduction is achieved after 1–2 months and though re‐evaluation could be performed at 1–2 months, most cases will benefit in waiting up to 3 months for a further 0.5–1 mm of PPD reduction. However, other evidence [8] has suggested that a longer period of approximately 12–15 weeks should be waited before re‐treating the diseased site in order to establish better tissue healing after an initial treatment and thus allow for better clinical outcomes of retreatment [9].

The superiority of MINST was particularly evident in the reduction of BoP‐positive sites with residual pockets and delta values of pockets with 4–5 mm PPD, consistent with previous findings evidencing that deep pockets respond more favorably to minimally invasive mechanical debridement compared with traditional CNST.

Moreover, the regression analysis evidenced that the MINST approach was able to significantly determine a greater PPD reduction across all follow‐up sessions compared to CNST, and that patient compliance and the number of diseased sites with PPD > 4 mm also BoP+ influenced significantly the PPD reduction, corroborating previous randomized controlled trials that demonstrated that MINST provides equivalent or superior outcomes compared to CNST in both initial and supportive therapy [8] and for retreatment pockets [9, 10].

The present trial presented some limitations. First, although the sample size was adequate to detect differences in PPD, the relatively small cohort and single‐centre design may limit generalizability. Although the results were assessed up to 12 months, a longer follow‐up would be needed to better confirm the effectiveness of treatments on periodontal outcomes and tooth survival. Third, the absence of patient‐reported outcomes represented another study limitation, as did the absence of a prospectively registered trial on clinicaltrials.gov.

## 5 Conclusions

In the present study, MINST was more effective than conventional CNST for retreating residual pockets in periodontitis patients with 12 months of follow‐up. Predictors of poorer response included high daily cigarette consumption, erratic or non‐compliant supportive care, high baseline FMBS, and a greater number of deep or high numbers of diseased sites.

## Funding

The study was supported by the Italian Minister of Health (project no. PNRR‐POC‐2023‐12 377 354), a grant from the Policlinic Hospital—University of Catania, Catania, Italy, under the head of Prof. G.I.

## Disclosure

*Artificial Intelligence Statement*: This manuscript did not use artificial intelligence in any capacity.

## Conflicts of Interest

The authors declare no conflicts of interest.

## Supporting information


**Figure S1:** Study flowchart.

**Table S1:** CONSORT Guidelines.

**Table S2:** Periodontal characteristics of the analyzed sample at baseline and at each follow‐up session of the active periodontal treatment performed with quadrant‐wise subgingival instrumentation comparison between follow‐up sessions.

**Table S3:** Clinical characteristics of the analyzed sample. BMI, Body Mass Index; KT, Keratinized Tissue.

**Table S4:** Periodontal parameters in both groups at baseline and at each follow‐up session.

**Table S5:** Variables that did not result in a significant effect from the mixed generalized linear regression model for PPD changes at all follow‐up sessions. Results were expressed as beta coefficient estimation (*β*), 95% Confidence Interval (95% CI) and *p*‐value. PPD, Probing Pocket Depth; CAL, Clinical Attachment Loss; KT, Keratinized tissues.

**Data S1:** jre70083‐sup‐0007‐Supplementary file 1.docx.

## Endnotes

^1^
UNC‐15, Hu‐Friedy, Milan, Italy.

^2^
Meridol, CP‐GABA, Hamburg, Germany.

^3^
Tepe, Malmo, Sweden.

^4^
Instrument PS EMS Electro Medical System S.A., Pero, Italy.

^5^
Satelec Ultrasonics, Acteon, Varese, Italy.
