# Supplementary material for: Minimally Invasive (MINST) Versus Conventional Non‐Surgical Therapy for Residual Pockets in Patients With Periodontitis: A Randomized Clinical Trial
Source: J Periodontal Res. 2026 Jun 18;61(9):1086–8. doi: 10.1111/jre.70083 (PMC13768984; doi:10.1111/jre.70083)
Supplement: Supplementary file 2 [file JRE-61-1086-s002.docx]

**Supplementary Table 5 :** Mixed generalized linear regression model for PPD changes at all follow-up sessions. For treatment, control was set as a reference; for gender, female was set as a reference; for gingival biotype, thick biotype was set as a reference; for patient compliance, regular supportive oral care was set as a reference. Results were expressed as beta coefficient estimation (β), 95% Confidence Interval (95% CI) and p-value. PPD, Probing Pocket Depth; FMBS, Full-Mouth Bleeding Score; PISA, Periodontal Inflamed Surface Area.

| **Parameter** | **Crude model** | | | **Adjusted model** | | |
| --- | --- | --- | --- | --- | --- | --- |
|  | **β** | **95% C.I.** | **P-value** | **β** | **95% C.I.** | **P value** |
| **Fixed effects** |  | | |  |  |  |
| *Treatment* | -3.39 | -4.126;-2.555 | 0.014 | -3.22 | -3.155;-1.574 | 0.023 |
| Cigarettes/day | 3.25 | 0.754; 4.515 | 0.018 | 4.25 | 1.874;6.225 | 0.032 |
| Patient compliance | 4.52 | 1.896;6.547 | 0.014 | 2.28 | 1.245;4.479 | 0.041 |
| no. sites PPD ≥ 4mm BoP+ | 2.59 | 1.025;4.236 | 0.006 | 2.36 | 1.458;4.562 | <0.001 |
| no. sites PPD 4-5 mm | 3.28 | 1.253;4.251 | 0.036 | 2.17 | 1.128;3.568 | 0.031 |
| no. sites PPD ≥ 6mm | 2.66 | 1.321;3.764 | 0.044 | 2.96 | 0.463;3.741 | 0.027 |
| % FMBS | 2.78 | 1.552;3.984 | 0.002 | 3.28 | 2.415;5.322 | 0.005 |
| PISA | 2.33 | 1.234;4.452 | 0.039 | 2.47 | 1.568;3.456 | 0.018 |
| **Random effects** |  | | |  |  |  |
| Number of diseased sites per patient | -2.19 | -2.953;-1.012 | 0.045 | -1.21 | -1.782;-0.691 | 0.048 |
