# Supplementary material for: Minimally Invasive (MINST) Versus Conventional Non‐Surgical Therapy for Residual Pockets in Patients With Periodontitis: A Randomized Clinical Trial
Source: J Periodontal Res. 2026 Jun 18;61(9):1086–8. doi: 10.1111/jre.70083 (PMC13768984; doi:10.1111/jre.70083)
Supplement: Supplementary file 3 [file JRE-61-1086-s005.docx]

**Supplementary Table 4:** Periodontal parameters in both groups at baseline and at each follow-up session.

| **Timepoints** | **Control group (n= 27)** | **Test group (n= 27)** | **p-value intergroup** |
| --- | --- | --- | --- |
|  | Median (IQR) | Median (IQR) |  |
| **PPD (mm)** | | | |
| Baseline | 5.17 (3.9-5.8) | 5.25 (3.8-6.2) | 0.341 |
| 1-months | 3.89 (3.1-4.3) | 3.23 (2.8-3.5) | 0.042 |
| 3-months | 3.78 (3.2-4.2) | 3.11 (2.5-3.8) | 0.047 |
| 6-months | 3.56 (2.9-3.9) | 3.14 (2.7.3.7) | 0.079 |
| 9-months | 3.78 (3-4.1) | 2.95 (2.7-3.4) | 0.015 |
| 12-months | 3.33 (2.7-3.6) | 2.73 (2.6-3.3) | 0.038 |
| Δ 0-12 months | 1.84 (1.6-2.3) | 2.52 (1.9-2.8) | 0.045 |
| Intragroup p-value | 0.041 | 0.007 |  |
| **No. Pockets PPD < 4mm** | | | |
| Baseline | - | - | - |
| 1-months | 49.6 (38.9-56.7) | 59.5 (45.6-64.2) | 0.048 |
| 3-months | 54.4 (46.2-63.4) | 64.6 (46.2-75.5) | 0.056 |
| 6-months | 56.5 (48.2-67.3) | 67.9 (52.4-79.2) | 0.061 |
| 9-months | 65.3 (57.2-75.6) | 79.1 (69.3-86.5) | 0.044 |
| 12-months | 61.3 (58.4-75.5) | 76.5 (65.9-84.5) | 0.036 |
| Δ 1-12 months | 22.9 (17.5-29.6) | 35.2 (24.2-41.3) | 0.040 |
| Intragroup p-value | 0.024 | 0.028 |  |
| **No. pockets PPD 4-5 mm** | | | |
| Baseline | 34.9 (27.5-38.5) | 36.5 (28.4-39.6) | 0.322 |
| 1-months | 29.6 (21.5-35.5) | 26.5 (21.2-31.4) | 0.087 |
| 3-months | 27.8 (19.4-33.2) | 24.1 (21.7-34.6) | 0.066 |
| 6-months | 29.5 (18.5-35.6) | 23.5 (17.9-28.6) | 0.052 |
| 9-months | 26.9 (21.3-36.7) | 20.4 (15.6-29.6) | 0.014 |
| 12-months | 26.7 (11.2-31.5) | 19.2 (14.6-26.5) | 0.032 |
| Δ 0-12 months | 8.2 (6.5-9.7) | 17.3 (11.5-24.8) | 0.006 |
| Intragroup p-value | 0.047 | 0.015 |  |
| **No. Pockets PPD ≥ 4mm BoP+** | | | |
| Baseline | 48.9 (36.7-57.5) | 50.5 (13.2-16.5) | 0.115 |
| 1-months | 39.5 (32.2-47.5) | 33.5 (28.9-38.5) | 0.067 |
| 3-months | 33.5 (28.9-40.2) | 31.5 (27.2-37.4) | 0.229 |
| 6-months | 29.5 (24.5-36.3) | 24.5 (20.3-31.2) | 0.055 |
| 9-months | 22.5 (18.5-26.4) | 18.4 (10.1-21.3) | 0.051 |
| 12-months | 18.6 (9.6-13.8) | 12.1 (8.9-15.6) | 0.023 |
| Δ 0-12 months | 30.3 (22.5-37.5) | 38.4 (27.5-41.4) | 0.009 |
| Intragroup p-value | 0.031 | 0.012 |  |
| **No. pockets PD 5-6 mm** | | | |
| Baseline | 21.3 (11.4-20.5) | 22.8 (15.5-26.5) | 0.123 |
| 1-months | 16.7 (9.1-17.5) | 14.5 (9.2-17.4) | 0.294 |
| 3-months | 16.4 (11.2-17.5) | 13.6 (9.6-17.4) | 0.104 |
| 6-months | 15.3 (10.5-17.4) | 13.5 (8.9-16.2) | 0.079 |
| 9-months | 14.9 (10.1-19.5) | 8.9 (7.1-14.3) | 0.011 |
| 12-months | 12.6 (9.1-18.3) | 10.2 (8.6-14.5) | 0.067 |
| Δ 0-12 months | 6.7 (3.1-8.4) | 12.6 (9.2-15.4) | 0.009 |
| Intragroup p-value | 0.045 | 0.031 |  |
| **No. pockets PPD ≥6 mm** | | | |
| Baseline | 11.4 (8.5-14.6) | 10.5 (7.2-13.9) | 0.255 |
| 1-months | 9.5 (5.5-13.1) | 9.1 (3.5-5.7) | 0.271 |
| 3-months | 8.6 (6.4-9.5) | 7.1 (5.1-8.3) | 0.098 |
| 6-months | 8.4 (6.1-8.9) | 6.5 (4.6-7.8) | 0.053 |
| 9-months | 7.2 (5.3-8.4) | 4.1 (2.9-5.1) | 0.041 |
| 12-months | 6.3 (4.5-7.6) | 4.3 (3.1-5.7) | 0.011 |
| Δ 0-12 months | 5.1 (3.5-8.4) | 6.2 (4.1-8.7) | 0.111 |
| Intragroup p-value | 0.048 | 0.044 |  |
