# Supplementary material for: Minimally Invasive (MINST) Versus Conventional Non‐Surgical Therapy for Residual Pockets in Patients With Periodontitis: A Randomized Clinical Trial
Source: J Periodontal Res. 2026 Jun 18;61(9):1086–8. doi: 10.1111/jre.70083 (PMC13768984; doi:10.1111/jre.70083)
Supplement: Supplementary file 4 [file JRE-61-1086-s006.docx]

**Supplementary Table S6:** Variables that did not result in a significant effect from the mixed generalised linear regression model for PPD changes at all follow-up sessions. Results were expressed as beta coefficient estimation (β), 95% Confidence Interval (95% CI) and p-value. PPD, Probing Pocket Depth; CAL, Clinical Attachment Loss; KT, Keratinized tissues.

| **Parameter** | **Crude model** | | | **Adjusted model** | | |
| --- | --- | --- | --- | --- | --- | --- |
|  | **β** | **95% C.I.** | **P-value** | **β** | **95% C.I.** | **P value** |
| **Fixed effects** |  | | |  |  |  |
| *Time* | 2.54 | -1.158;4,122 | 0.054 | - | - | - |
| Treatment*Time | -3.24 | -4.578;1.546 | 0.556 | - | - | - |
| Age | -2.22 | -4.239;-1.856 | 0.036 | -0.19 | -1.455;1.106 | 0.332 |
| Gender | 1.12 | -1.571;2.455 | 0.126 | - | - | - |
| Smoking | 2.14 | 0.644;3.274 | 0.009 | 3.79 | -1.566;4.528 | 0.075 |
| Gingival biotype | -0.74 | -2.447;-0.595 | 0.025 | -1.58 | -2.458;1.466 | 0.258 |
| no. sites PPD < 4mm | -3.15 | -6.541;-2.456 | 0.012 | -1.36 | -6.261;1.569 | 0.214 |
| Median CAL | 2.14 | 1.559;2.958 | 0.044 | 2.12 | -0.124;3.751 | 0.128 |
| KT | -1.39 | -3.459;0.441 | 0.129 | - | - | - |
| **Random effects** |  | | |  |  |  |
| Patient | 2.34 | -1.323;3.326 | 0.331 | 1.12 | -0.455;2.456 | 0.114 |
