# Supplementary material for: Minimally Invasive (MINST) Versus Conventional Non‐Surgical Therapy for Residual Pockets in Patients With Periodontitis: A Randomized Clinical Trial
Source: J Periodontal Res. 2026 Jun 18;61(9):1086–8. doi: 10.1111/jre.70083 (PMC13768984; doi:10.1111/jre.70083)
Supplement: Supplementary file 5 [file JRE-61-1086-s007.docx]

**Supplementary Table S2:** Clinical sample characteristics. BMI, Body Mass Index; KT, Keratinized Tissue.

| **Parameter** | **Control group (n= 27)** | **Test group (n= 27)** | **P value** |
| --- | --- | --- | --- |
| Gender (male), no. (%) | 15 (55.5) | 17 (63) | 0.245 |
| Age, median (IQR) | 49.5 (45.7;57.1) | 50.4 (46.4;56.5) | 0.335 |
| Race, Caucasian, no. (%) | 27 (100) | 21 (100) | 0.847 |
| Patient’s education level, no. (%) |  |  | 0.314 |
| *Primary School* | 11 (40.7) | 10 (37) |  |
| *High School* | 12 (44.5) | 14 (51.9) |  |
| *University* | 4 (14.8) | 3 (11.1) |  |
| BMI (kg/m^2^), median (IQR) | 20.9 (17.5-23.2) | 21.3 (16.8-24.6) | 0.219 |
| Patient’s compliance, no. (%) |  |  |  |
| *Regular* | 17 (63) | 16 (59.3) | 0.783 |
| *Erratic* | 6 (22.2) | 8 (29.6) | 0.538 |
| *Non compliers* | 4 (14.8) | 3 (11.1) | 0.688 |
| Smoking, no. (%) |  |  |  |
| *Never smokers* | 16 (59.3) | 14 (51.9) | 0.587 |
| *Ex-smokers* | 6 (22.2) | 7 (26) | 0.746 |
| *Current smokers* | 5 (18.5) | 6 (22.2) | 0.738 |
| No. cigarettes/day, median (IQR) | 10.5 (7.8-11.7) | 11.1 (8.3-13.7) | 0.551 |
| Stage and grade periodontitis, no. (%) |  |  |  |
| Stage III periodontitis | 16 (59.3) | 14 (51.9) | 0.287 |
| *Grade A* | 8 (29.6) | 7 (26) | 0.551 |
| *Grade B* | 5 (18.5) | 5 (18.5) | 0.467 |
| *Grade C* | 3 (11.1) | 2 (7.4) | 0.298 |
| Stage IV periodontitis | 11 (40.7) | 13 (48.1) | 0.657 |
| *Grade A* | 6 (22.2) | 7 (26) | 0.714 |
| *Grade B* | 3 (11.1) | 4 (14.8) | 0.329 |
| *Grade C* | 2 (7.4) | 3 (11.1) | 0.557 |
| Extent of periodontitis, no. (%) |  |  |  |
| *Localized (<30% of teeth involved)* | 12 (44.5) | 10 (37) | 0.578 |
| *Generalized (>30% of teeth involved)* | 15 (55.5) | 17 (63) | 0.579 |
| Number of teeth, median (IQR) | 21.5 (18.6-25.8) | 22.3 (19.1-24.7) | 0.144 |
| Type of teeth |  |  |  |
| *Incisor* | 7 | 9 | 0.098 |
| *Canine* | 7 | 5 | 0.135 |
| *Premolar* | 10 | 8 | 0.224 |
| *Molar* | 5 | 8 | 0.146 |
| Site locations |  |  |  |
| *Maxilla* | 16 | 14 | 0.067 |
| *Mandible* | 13 | 16 | 0.104 |
| *Buccal* | 17 | 14 | 0.201 |
| *Lingual* | 12 | 16 | 0.068 |
| *Mesial buccal* | 7 | 6 | 0.689 |
| *Mesial lingual* | 5 | 7 | 0.567 |
| *Distal buccal* | 10 | 8 | 0.445 |
| *Distal lingual* | 7 | 9 | 0.291 |
| Gingival biotype, no. (%) |  |  |  |
| *Thin biotype* | 15 (55.5) | 14 (51.9) | 0.842 |
| *Thick biotype* | 12 (44.5) | 13 (48.1) | 0.611 |
| KT, mm, Median (IQR) | 2.49 (2.07-2.95) | 2.53 (2.17-2.86) | 0.551 |
